# Acute esophageal tissue reaction after Cryoballoon ablation in patients with paroxysmal atrial fibrillation - a LGE-MRI Study

**DOI:** 10.1186/1532-429X-16-S1-P142

**Published:** 2014-01-16

**Authors:** Christian Mahnkopf, Marcel Mitlacher, Philipp Halbfass, Oliver Turschner, Johannes Brachmann

**Affiliations:** 1 Department of Cardiology, Klinikum Coburg, Coburg, Germany

## Background

Esophageal wall thermal injury after Cryoballoon ablation is a potentially serious complication. Late-Gadolinium Enhancement MRI (LGE-MRI) allows an accurate and non-invasive detection of acute tissue reaction. We thought to compare the relationsship of acute esophageal tissue reaction detected using LGE-MRI acute after pulmonary vein isolation (PVI) with the Arctic Front (AF) and the Artic Front Advanced (AFA) Cryoballoon in patients with paroxysmal atrial fibrillation (PAF).

## Methods

Fifty-six patients with PAF (35 male, mean age 59.9 ± 10.5 years old) were included into this study. All patients underwent PVI using the Cryoballoon technique. The traditional AF balloon was used in 33 patients (58.9%) whereas the new AFA was used in 23 patients (41.1%). LGE-MRI (MRI Verio 3T, Siemens, Erlangen, Germany) of the left atrium (LA) was performed before and within 24 hours after PVI in all patients.

## Results

**Figure 1:**
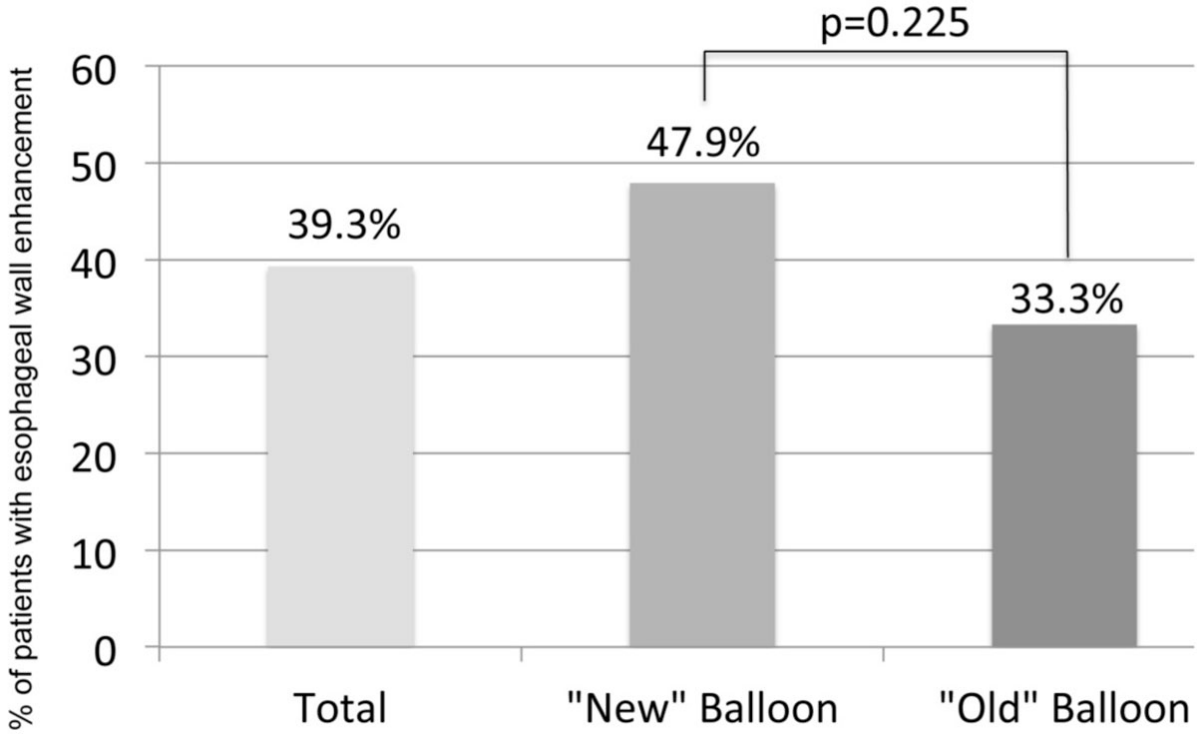
**Columns gives % of patients found with esophageal wall enhancement acute after pulmonary vein isolation using the "old" Artic Front or the "new" Artic Front Advanced Balloon**.

**Figure 2:**
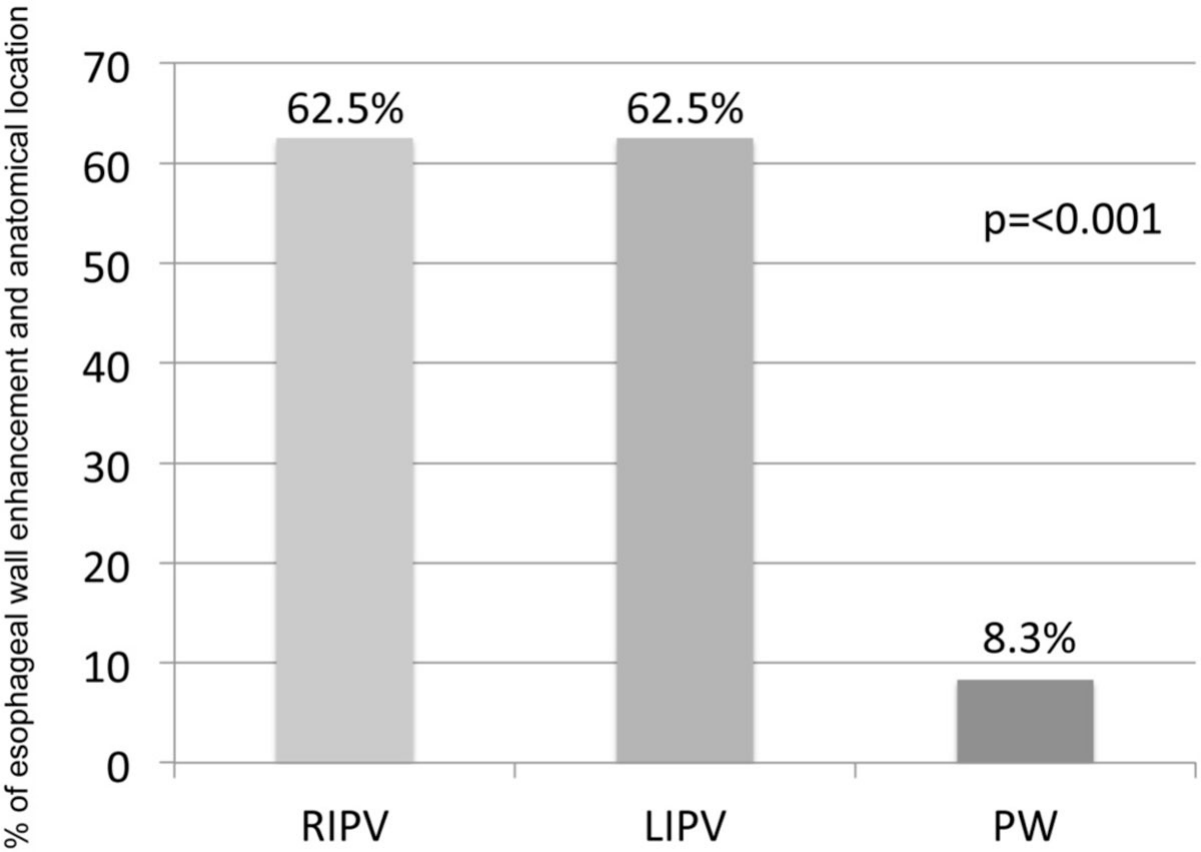
**Columns gives % of patients with esophageal wall enhancement acute after pulmonary vein isolation regarding to the anatomical location and contact of the esophagus with the left atrium**. RIPV = Right inferior pulmonary vein. LIPV = Left inferior pulmonary vein PW = Posterior Wall.

**Figure 3:**
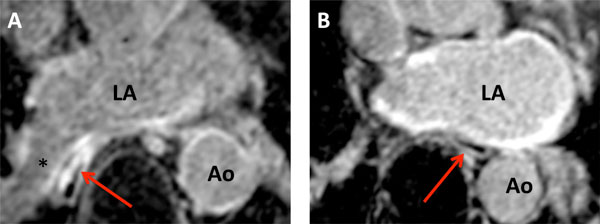
**Example of a patient with (A) and without (B) esophagus wall enhancement (red arrow) acute after cryoballoon ablation**. Note the anatomical proximity of the esophagus to the right inferior pulmonary vein in Figure 3A and the relationship between the esophagus and the posterior wall in Figure 3B. * = Right inferior pulmonary vein; Ao - Aorta; LA - Left atrium.

Significant enhancement of the esophageal wall (EWE) as a sign of acute tissue reaction after ablation was found in 22 patients (39.3%). Local tissue reaction was independent from the type of Cryoballoon as EWE was detected in 33.33% in the AF and 47.87% of the AFA group (p = 0.275, Figure 1). EWE was related to the anatomical conditions of the esophagus as EWE was significant higher in those patients where the esophagus was in contact with the left or right inferior pulmonary veins compared to patients where the esophagus was in contact with the posterior wall of the LA (62.5% vs. 8.3%, p = < 0.001; Figure 2 and Figure 3). Anatomical location of the esophagus was unchanged in all patients comparing the pre and acute LGE-MRI.

## Conclusions

From our preliminary results, acute esophageal tissue reaction detected using LGE-MRI after Cryoballoon ablation is correlated with the anatomical relationship between the esophagus and the left atrium and is independent form the used Cryoballoon. Anatomical location of the esophagus can be easily assed with the MRI and should be considered for patients safety during the ablation procedure.

## Funding

None.

